# Knowledge and attitude towards rape and child sexual abuse – a community-based cross-sectional study in Rural Tanzania

**DOI:** 10.1186/s12889-015-1757-7

**Published:** 2015-04-28

**Authors:** Muzdalifat Abeid, Projestine Muganyizi, Siriel Massawe, Rose Mpembeni, Elisabeth Darj, Pia Axemo

**Affiliations:** Department of Women’s and Children’s Health, International Maternal and Child Health (IMCH), Uppsala University, Uppsala, SE-75185 Sweden; Department of Obstetrics/Gynecology, Muhimbili University of Health and Allied Sciences (MUHAS), Dar es Salaam, P.O.Box 65117, Tanzania; Department of Epidemiology and Biostatistics, Muhimbili University of Health and Allied Sciences (MUHAS), Dar es Salaam, P.O.Box 65117, Tanzania; Department of Public Health and General Practice, Norwegian University of Science and Technology, Trondheim, P.O.Box 8905, Norway

**Keywords:** Attitude, Child sexual abuse, Community, Knowledge, Rape, Sexual violence

## Abstract

**Background:**

Violence against women and children is globally recognized as a social and human rights concern. In Tanzania, sexual violence towards women and children is a public health problem. The aim of this study was to determine community knowledge of and attitudes towards rape and child sexual abuse, and assess associations between knowledge and attitudes and socio-demographic characteristics.

**Methods:**

A cross-sectional study was undertaken between May and June 2012. The study was conducted in the Kilombero and Ulanga rural districts in the Morogoro Region of Tanzania. Men and women aged 18–49 years were eligible for the study. Through a three-stage cluster sampling strategy, a household survey was conducted using a structured questionnaire. The questionnaire included socio-demographic characteristics, attitudes about gender roles and violence, and knowledge on health consequences of rape. Data were analyzed using the Statistical Package for Social Sciences (SPSS) software, version 21. Main outcome measures were knowledge of and attitudes towards sexual violence. Multivariate analyses were used to assess associations between socio-demographic characteristics and knowledge of and attitudes towards sexual violence.

**Results:**

A total of 1,568 participants were interviewed. The majority (58.4%) of participants were women. Most (58.3%) of the women respondents had poor knowledge on sexual violence and 63.8% had accepting attitudes towards sexual violence. Those who were married were significantly more likely to have good knowledge on sexual violence compared to the divorced/separated group (AOR = 1.6 (95% CI: 1.1-2.2)) but less likely to have non-accepting attitudes towards sexual violence compared to the single group (AOR = 1.8 (95%CI: 1.4-2.3)). Sex of respondents, age, marital status and level of education were associated with knowledge and attitudes towards sexual violence.

**Conclusions:**

Our study showed that these rural communities have poor knowledge on sexual violence and have accepting attitudes towards sexual violence. Increasing age and higher education were associated with better knowledge and less accepting attitudes towards sexual violence. The findings have potentially important implications for interventions aimed at preventing violence. The results highlight the challenges associated with changing attitudes towards sexual violence, particularly as the highest levels of support for such violence were found among women.

## Background

Violence against women and children is increasingly recognized as a specific health and human rights issue. Studies across continents indicate a high prevalence of rape and child sexual abuse (CSA) [1-5]. Sexual violence against women and children is closely related to the social and cultural context [6-8]. Globally, the prevalence of CSA varies between 2 and 62% for girls and 3 and 16% for boys, depending on setting and definition used [9]. Rape prevalence estimations are faced by methodological challenges and definition differences used by various studies. However, in studies worldwide it is estimated that between 14 and 25% of adult women have been raped during their lifetime [5,8,10,11]. This study focuses on rape against women and children, for which we used the term “sexual violence”. Rape was defined as sexual contact that occurs without the victim’s consent, involves the use of force, threat of force, intimidation, or when the victim is of unsound mind due to illness or intoxication and involves sexual penetration of the victim’s vagina, mouth or rectum [3,12,13].

Rape against women is a public health problem in Tanzania, and it is estimated that about 20% of adult women in an urban setting have experienced a completed rape [14]. Between 1995 and 2007, the number of rape incidents reported annually to the Ministry of Home Affairs (2009) increased from less than 1,000 to 4,500 [15]. The National Survey on Violence Against Children (VAC) in Tanzania provides national estimates of the magnitude and nature of sexual, physical, and emotional violence experienced by girls and boys in Tanzania. The estimates show that violence against children is so serious that, at the age of 18, more than a quarter of girls (28%) and 13% of boys have experienced sexual violence [16].

Rape is associated with a range of reproductive health consequences. Some of these consequences are direct, such as acute injuries, sexually transmitted infections (STIs) including HIV, and unwanted pregnancies [5,11]. Emotionally, the problem is associated with chronic somatic disorders, anxiety, depression, high risk sexual behavior, chronic illnesses and socio-economic consequences that generally impact negatively on the victim’s quality of life [17].

Studies in low- and middle-income countries have demonstrated high prevalence of intimate partner violence and its determinants [7,11,18-21]. The WHO multi-country study indicates that violence is also common in intimate relationships in Tanzania where it is estimated that 31% of women in the rural and 23% in the urban areas have experienced sexual violence during their lifetime [5]. The same study, in a 12-month prevalence survey, also estimated that 18% of the women in the rural areas of Tanzania have experienced sexual violence [5]. These results are supported by another population-based study from the northern part of Tanzania where lifetime and 12-month prevalence of physical or sexual violence was reported to be 26% and 21% respectively [11]. In view of the magnitude of violence against women in Tanzania, the WHO suggests a comprehensive health sector response providing adequate, non-stigmatizing and supportive services for intimate partner violence survivors [5].

Previous studies in rural communities of Sub-Saharan Africa highlight the presence of marked limited services, higher poverty rates and often low literacy rates [22,23]. The literature shows that women’s educational level exerts a protective effect with regard to domestic violence [19,24]. The reason behind this observation may be because education increases autonomy and economic empowerment [6,25]. However, some studies found little association between education level and exposure to violence [26]. In community- and hospital-based studies among community groups and health care workers in Tanzania, it was found that apart from seeking medical and legal care, rape survivors who report to local government leaders and health facilities often do so in order to obtain opinions on legal or social procedures to follow and to establish the seriousness of the events [27-29]. It was further shown that the consequences of rape and CSA are not well understood by the community, including health care workers [27-29]. It has been shown that community reactions to these victims are important predictors of the long-term consequences of rape and CSA [28,30,31]. These findings are indicative of barriers to adequate care of rape and CSA survivors, including the identification of intimate partner violence victims, in the community and at health facilities where the victims are most likely to be seen early enough for effective initiation of emergency care.

Other barriers to seeking care include rape myths. Rape myths are those ideas or beliefs that “deny or minimize victim injury or blame the victims for their own victimization” [32]. Rape myths that are commonly accepted include, the woman deserved to be raped; she asked for it through her provocative behavior or dress; there was not much physical damage so it was not rape; stranger rape is more prevalent than acquaintance rape; and, a woman cannot be raped by her husband [32-35]. There is a growing body of evidence showing that despite years of public education about sexual violence, rape myths and gender stereotypes are still accepted, believed and propagated by communities [32-35]. In societies with high prevalence of interpersonal violence, attitudes that tolerate violence against women are viewed as normative behavior [6,36]. Exploring the community’s knowledge of and attitudes towards sexual violence in rural settings is of the utmost importance to enable the development of interventions relevant to the entire country. Currently very few population-based studies have focused on the underlying factors related to positive attitude towards violence against women [37-39]. The aim of this study was to determine community knowledge of and attitudes towards sexual violence, and assess their association with socio-demographic characteristics.

## Methods

### Study design

A cross-sectional, multi-stage, random sample survey study was undertaken in May and June 2012.

### Setting

The study was conducted in the Kilombero and Ulanga districts in the Morogoro Region of Tanzania. Morogoro is situated about 350 km south-west of Dar es Salaam. These are rural districts with the main economic activities being farming and small-scale fishing. Initially, Kilombero and Ulanga was one district and these two districts have comparable socio- demographic characteristics, economic activities and health system organization. The two districts are separated by the Kilombero River. The Kilombero district has 5 divisions, 19 wards, 81 villages and 365 hamlets with a total population of 416,401 [40]. It has an organized health system, with one designated district hospital, one private hospital, 5 health centers (one in each division) and 38 dispensaries. This district is unique in that it has large commercial sugar cane farms that are owned by the biggest sugar factories in Tanzania. These factories are also situated in the district and employ laborers who are recruited from different parts of the country. The Ulanga district has a total population of 234,219, and comprises 7 divisions, 31 wards, 91 villages and 40 health facilities with two hospitals, one of which is a district hospital [41]. The study involved two administrative divisions, one from the Kilombero district (Mngeta division), and one from the Ulanga district (Mwaya division), both together encompassing approximately 150,000 inhabitants. The literacy rate in the Morogoro region is 85% for men and 73% for women. Only 12% of women and 14% of men had completed some primary level of schooling [42].

### Participants

Participants were made up of both men and women who were eligible for inclusion if they were between the ages of 18 and 49 years, had lived in the village for at least a year, and if they usually shared meals with others in the household.

### Sample

A multi-stage random sample was selected independently in each district; in the first stage we randomly selected one division (Mngeta) out of five in Kilombero, and one (Mwaya) out of seven in Ulanga. In the Mngeta division there are 2 wards and 6 villages; likewise in the Mwaya division. Therefore, in the second stage we randomly selected one ward in each division, and in the third stage one village in each ward was selected. Thus we selected two villages with an average of 6,000 individuals in each, that is, in total, around 12,000 individuals. Assuming a high negative attitude towards gender violence of 70% [26], confidence limits of 5%, and a design effect for cluster survey of 2.0, a sample size of 1,082 would be enough to obtain a 99% confidence level that the results could be generalized to the wider population. We included all the households within each village in the survey. A household selection form was used to ascertain whether the selected household had any members who were eligible to complete the community survey. The head of the household was asked to list all household members. Eligible members were shortlisted and then, using a ballot technique, one eligible member was selected. In order to ensure the safety and confidentiality of the respondents, a maximum of one person per household was selected to complete the survey. At least three repeat visits were made to households where respondents were not available at the time of the first visit.

### Data collection and measures

A pilot-tested and standardized baseline questionnaire was used. It elicited data on socio- demographics characteristics, attitudes toward gender stereotypes and rape myths, health consequences of rape, and radio ownership. The questionnaire was initially prepared in English and then translated into Swahili, the national language of Tanzania. Questions on violence acceptability were adapted from those used in the WHO multi-country study on women’s health and domestic violence [5] and questions on rape myths were adapted from the Attitude Rape Victim Scale (ARVS) which was proven to be culturally appropriate [43]. A total of ten field workers, two men and eight women, who had either nursing or assistant medical officer backgrounds and who were not inhabitants of the study divisions, were selected and trained in the standardized use of the questionnaire, the nature of the study, ethical issues related to this study and techniques to conduct such sensitive interviews [44,45]. The survey was conducted with the support of local leaders who assisted in introducing the research assistants to the household members. The field supervisor (MA) was easily accessible by the research assistants through mobile phone or physically when necessary.

The study was then introduced to the eligible household member as *“****The study of women’s health issues”***. Verbally informed consent was then sought. Interviews were conducted in the home which was the preferred location for all respondents. Each interview lasted for 45 minutes on average. All completed questionnaires were checked by the field supervisor, who is the first author (MA), upon completion, and where problems were identified, the questionnaire was returned to the interviewer for corrections or for missing information to be completed. Regular debriefing meetings were scheduled to enable the research team to discuss their feelings related to the study and to explore how it was affecting them with an aim to reduce the stress of the field work and avert any negative consequences [44,45]. All interviews ended with the researchers reinforcing that the participation of the household members was voluntary, and reminding them that the information they had shared was important and would be used to help women.

### Measurements

The respondents’ knowledge on sexual violence was tested in their answers to the following 5 questions: *circumstances that influence rape*; *complications of rape/CSA*; *medical care of rape/CSA survivors at health facility*; *perpetrators of rape/CSA*; and *the Sexual Offence Act of Tanzania*, specifically, the minimum punishment for perpetrators of sexual violence. The 5 questions had a total of 25 correct responses and each correct answer was scored 1, thus, the maximum score was 25. The scale was dichotomized using the two-third rule to categorize respondents, with a score of 0-66% as having poor knowledge on sexual violence, and all those who scored 67-100% as having good knowledge on sexual violence (Table 1). Associations between social demographic characteristics and knowledge were then assessed.

**Table 1:** **Measurement of knowledge and attitudes toward sexual violence**

**Measurement**	**Criteria for labeling**
Knowledge	
(1) Causes of sexual violence	The total score was 25. Respondents with score 0-66% was labeled as having poor knowledge on sexual violence and coded as 0, and all those who score 67-100% labeled as having good knowledge on sexual violence and coded as 1.
- Effects of alcohol/illicit drugs	
- Effects of pornographic films	
- Changes in our culture	
(2) Consequences of sexual violence	
- Health and physical effects	
- Mental and psychological effects	
- Reproductive health effects	
- Long term effect on the victim’s development	
(3) Perpetrators of sexual violence	
- Not known	
- Strangers in the community	
- Close friends	
- Close relatives	
(4) Sexual offense Special Provision Act (SOSPA) for Tanzania	
- Number of years of imprisonment for perpetrators	
(5) Expected services at the health facility	
- Contraception, HIV/AIDS prophylaxis, STI treatment, wound care, psychotherapy,legal verification	
Attitudes favoring male dominance	The maximum score for all 14 statements is 28. Respondent not believing in most of the statements and scored 21–28 was labeled as *‘non-accepting’* coded as 1. Respondents scoring less than 21 were labeled as *‘accepting’* coded as 0.
(1) A man should show he is head of household	
(2) A decent wife obeys his husband	
(3) A wife is obliged to have sex with her husband	
(4) Marital disputes should not be exposed outside	
(5) Husband disciplines the wife by beating her	
Opinions on justifying husband beating his wife	
(1) Reason to hit: wife does not fulfill household duties	
(2) Reason to hit: wife refuses sex	
(3) Reason to hit: wife opposes his views/opinions	
(4) Reason to hit: wife is unfaithful	
(5) Reason to hit: wife is alcohol/drug abuse	
(6) Reason to hit: wife insults/disrespect	
Rape Myths	
(1) Reason women and girls are raped: the way they dress or act	
(2) Reason women and girls are raped: the place they work (bar, clubs, prostitute)	
(3) Reason women and girls are raped: they walk alone at night	

Attitude towards sexual violence was assessed in terms of *gender roles and acceptability of violence in the community and the rape myths:* respondents who believe that “men are justified in beating their wives under certain circumstances” (measured using six statements); and respondents who believe that “male dominance is appropriate” (measured using five statements). In exploring the community’s endorsement of rape myths, respondents were asked if it is the woman/girl’s fault that she was raped (measured using three statements). Responses that were either “agree” or “don’t know” to any statement scored 1, and responses that were “disagree” to any statement scored 2. The total possible score was 28 and all those who scored 21or more were considered as having a *non-accepting attitude*, and all those who scored less than 21were considered as having an *accepting attitude* (Table 1). Associations between social demographic characteristics and attitude were then assessed.

### Analysis

Data were entered twice for validation and this was completed using Epidata 3.1 [46]. Data analysis was performed using Statistical Package for Social Sciences (SPSS) software, version 21. A multilevel analysis was done using complex sample methods where design-based weights for different sampling strata were calculated as the reciprocal of the probability of selection of units in that particular strata. We took into account relationships across and within hierarchical strata of multi-stage design and variability at different strata. The analysis provided exact standard errors which took into account stratification and clustering. The dependent (outcome) variables were knowledge on sexual violence (poor/good) and attitude towards sexual violence (accepting/non-accepting). Bivariate analysis was performed to determine associations of participant characteristics with knowledge and attitude outcomes. Multiple logistic regression analyses were performed in a stepwise backward regression model to select variables for the final multivariate regression analysis model. Only variables with *p* < 0.2 were entered in the final model. Adjusted Odds Ratios and 95% Confidence Intervals were obtained to determine variables that independently predicted knowledge and attitudes towards rape and child sexual abuse. In all the analyses, a *p*-value of <0.05 was considered statistically significant.

### Ethical consideration

Ethical clearance to conduct this study was obtained from the ethical committee of the Muhimbili University of Health and Allied Sciences (MUHAS). Permission to conduct this study in the area was sought from the Kilombero and Ulanga districts authorities. We closely followed the ethical guidelines of research on violence against women approved by the WHO (2001) and the WHO/CIOMS’s (2002) ethical guidelines for biomedical research involving human subjects [47,48]. This implied asking for informed verbal consent after the participants have received an oral explanation of the goals and objectives of the study, its confidentiality safeguards and the potential risks and benefits of their voluntary participation. To ensure safety and confidentiality of participants, only one person per household was randomly selected to be interviewed. Interviews were conducted in a secluded spot in the participants’ home environment. At the end of the interview participants received contact details of the study coordinator, whom they could contact in case of distress. The questionnaires did not bear participants’ names and were locked in a safe, accessible only to the research team.

## Results

A total of 1,568 participants were interviewed. The response rate was 99.3%. In total, 915 (58.4%) of respondents were women. The mean age of respondents was 31 ± 7.4 years. More than 80% had completed primary education. Most (62.2%) respondents were married or cohabiting. Farming was the dominant occupation in the two districts and employed 87.4% of the study population. About 77% of respondents possessed a radio, as shown in Table 2.

**Table 2:** **Socio-demographic characteristics of respondents**

**Characteristic**	**Number (N = 1,568)**	**Percent (%)**
**Sex**	915	58.4
Female	653	41.6
Male		
**Age group (in years)**		
15 – 24	410	26.1
25 – 34	595	37.9
35 – 49	563	35.9
**Educational level**		
No formal education	142	9.1
Primary Education	1,308	83.4
Secondary Education and above	118	7.5
**Marital status**		
Married/cohabiting	969	62.2
Single	444	27.7
Divorced/separated/widowed	155	10.1
**Occupation**		
Student	56	3.6
Salaried employed	36	2.3
Self-employed	76	4.8
Peasant/farmer	1,370	87.4
Others	30	1.9
**Radio ownership**		
Yes	1,211	77.2
No	357	22.8

Men were found to have better knowledge of sexual violence than women (*p*-value < 0.001), as shown in Table 3*.* Having a higher level of education was also associated with a higher level of knowledge on sexual violence (*p*-value <0.001). Those who owned a radio had significantly better knowledge on sexual violence as compared to those without a radio (*p* < 0.040). The details are as shown in Table 3.

**Table 3:** **Descriptive statistics of knowledge towards sexual violence among respondents of rural Morogoro**

**Characteristics**	**Total**	**Good knowledge n (%)**		**Chi** ^**2**^
		**Yes**	**No**	***P*** **-value**
**Sex**				<0.001
Female	915	382 (41.7%)	533 (58.3%)	
Male	653	339 (51.9%)	314 (48.1%)	
**Age groups (in years)**				0.063
15 – 24	410	177 (43.2%)	233 (56.8%)	
25 – 34	595	263 (44.2%)	332 (55.8%)	
35 – 49	563	281 (49.9%)	282 (50.1%)	
**Educational level**				<0.001
No formal Education	142	44 (31.0%)	98 (69.0%)	
Primary Education	1,308	608 (46.5%)	700 (53.5%)	
Secondary Education and above	118	69 (58.5%)	49 (41.0%)	
**Marital status**				0.007
Married/cohabiting	969	467 (48.2%)	502 (51.8%)	
Single	444	200 (45.0%)	244 (55.0%)	
Divorced/separated/widowed	155	54 (34.8%)	101 (65.2%)	
**Occupation**				0.168
Unemployed	1,456	662 (45.5%)	794 (54.5%)	
Employed	112	59 (52.7%)	53 (47.3%)	
**Radio ownership**				0.040
Yes	1,211	574 (47.4%)	637 (52.6%)	
No	357	147 (41.2%)	210 (58.8%)	

**Figure 1:**
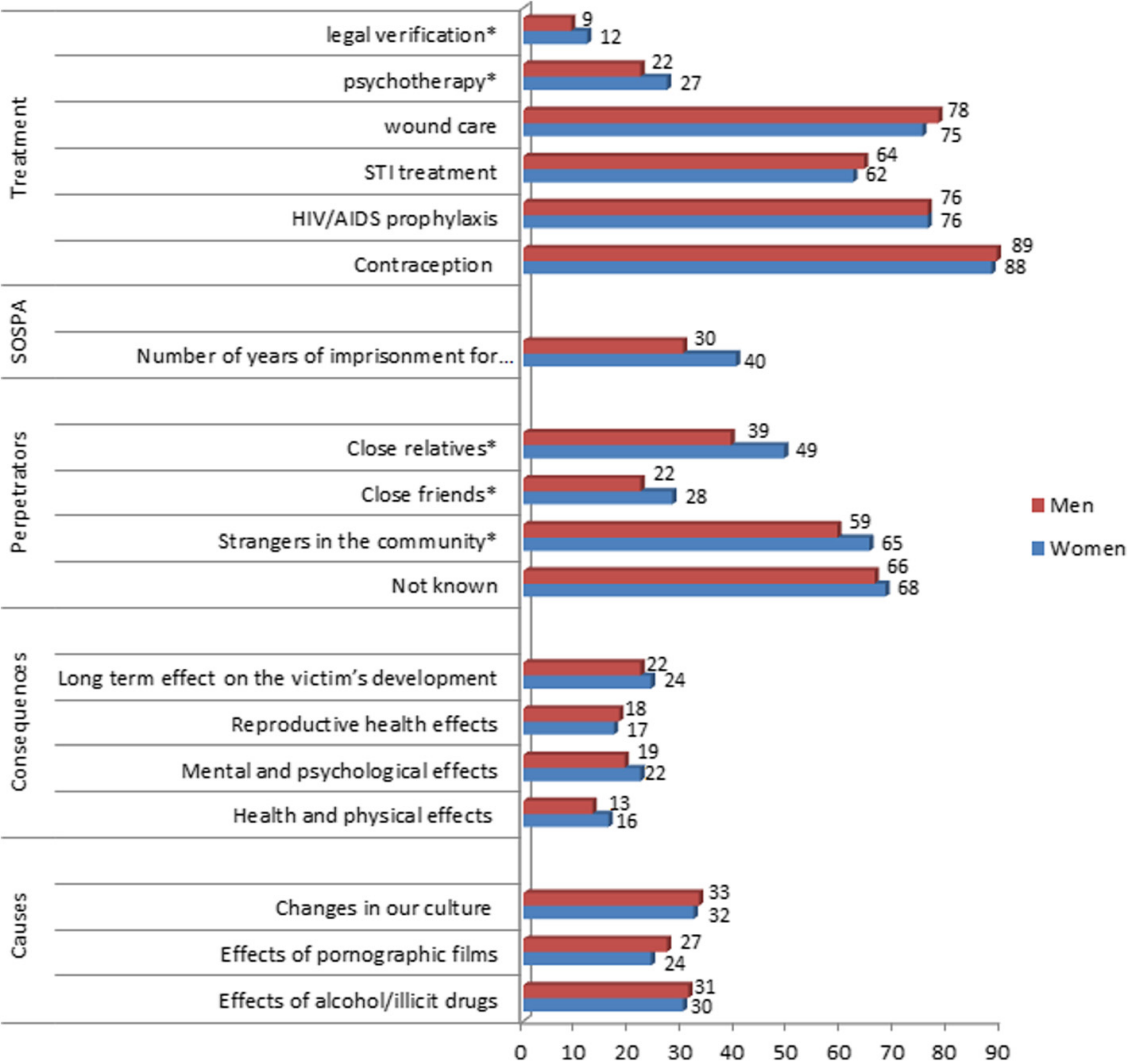
Gender analysis of knowledge on sexual violence. Percentage of all women and men aged 18–49 who do not know about the circumstances that influence sexual violence, the consequences of sexual violence, the perpetrators of violence, the sexual offence special provision act (SOSPA), and the medical treatment of sexual violence.

Figure 1 compares knowledge of sexual violence by gender of the respondents. Women were less knowledgeable on the consequences of sexual violence, perpetrators of violence and the law that convicts the perpetrators − the Sexual Offense Special Provision Act (SOSPA). Men, on the other hand, were less knowledgeable on the circumstances that influence sexual violence and the treatment that may be offered at the health facility.

### Factors associated with knowledge on sexual violence

All socio-demographic variables were associated with knowledge on sexual violence during bivariate analysis. After adjusting for other variables, occupation and radio ownership did not seem to be associated with knowledge on sexual violence; the rest persisted to be significantly associated. The older age group were more likely to have good knowledge on sexual violence [AOR = 1.4 (95% CI:1.0-1.8)] compared to younger age groups. The higher the education level, the more likely the participant was to have good knowledge on sexual violence [AOR = 3.1(95% CI:1.8 5.3)]. Those who were single, divorced or separated were significantly less likely to have good knowledge on sexual violence compared to the married/cohabiting group, as shown in Table 4.

**Table 4:** **Bivariate and multivariate logistic regression analysis of knowledge on sexual violence among respondents of rural Morogoro**

**Predictors**	**Number (N = 1,568)**	**Good knowledge n (%)**	Crude OR*	**95% confidence interval (CI)**	Adjusted OR**	**95% confidence interval (CI)**
**Sex**						
Female	915	382 (41.7%)	Ref		Ref	
Male	653	339 (51.9%)	1.5	(1.2-1.8)	1.3	(1.1-1.6)
**Age (in years)**						
15 – 24	410	177 (43.2%)	Ref		Ref	
25 – 34	595	263 (44.2%)	1.0	(0.8-1.3)	1.1	(0.8-1.4)
35 – 49	563	281 (49.9%)	1.3	(1.0-1.7)	1.4	(1.0-1.8)
**Educational level**						
No formal Education	142	44 (31.0%)	Ref		Ref	
Primary Education	1,308	608 (46.5%)	1.9	(1.3-2.8)	1.8	(1.2-2.6)
Secondary Education and above	118	69 (58.5%)	3.1	(1.9-5.2)	3.1	(1.8-5.3)
**Marital status**						
Divorced/separated/widow	155	54 (34.8%)	Ref		Ref	
Married/cohabiting	969	467 (48.2%)	1.7	(1.2-2.5)	1.6	(1.1-2.2)
Single	444	200 (45.0%)	1.5	(1.0-2.2)	1.3	(0.9-2.0)
**Occupation**						
Unemployed	1,456	662 (45.5%)	Ref		Ref	
Employed	112	59 (52.7%)	1.3	(0.9-2.0)	1.2	(0.8-1.8)
**Radio ownership**						
No	357	147 (41.2%)	Ref		Ref	
Yes	1,211	574 (47.4%)	1.3	(1.0-1.6)	1.2	(0.9-1.5)

OR - Odds Ratio.

*Bivariate logistic regression.

**Multiple logistic regression: age, sex, education, marital status, occupation and radio ownership were included.

Sex, education level, marital status and occupation influenced the attitudes towards sexual violence in bivariate analysis. The higher the level of education, the more likely the person was to have a non-accepting attitude towards sexual violence (*p*-value <0.001). Furthermore, a non-accepting attitude was more likely to be found among male respondents (*p*-value <0.001) and among participants who were employed (*p*-value 0.037). Other details are as shown in Table 5.

**Table 5:** **Descriptive statistics of acceptance of sexual violence among respondents of rural Morogoro**

**Characteristics**	**Total**	**Acceptance of sexual violence n (%)**		**Chi** ^**2**^
		**No**	**Yes**	***P*** **-value**
**Sex**				<0.001
Female	915	331 (36.2%)	584 (63.8%)	
Male	653	328 (50.2%)	325 (49.8%)	
**Age groups (in years)**				0.164
15 – 24	410	162 (39.5%)	248 (60.5%)	
25 – 34	595	243 (40.8%)	352 (59.2%)	
35 – 49	563	254 (45.1%)	309 (54.9%)	
**Educational level**				<0.001
No formal Education	142	46 (32.4%)	96 (67.6%)	
Primary Education	1,308	542 (41.4%)	766 (58.6%)	
Secondary Education and above	118	71 (60.2%)	47 (39.8%)	
**Marital status**				< 0.001
Married/cohabiting	969	358 (36.9%)	611 (63.1%)	
Single	444	229 (51.6%)	215 (48.4%)	
Divorced/separated/widowed	155	72 (46.5%)	83 (53.5%)	
**Occupation**				0.037
Unemployed	1,456	601 (41.3%)	855 (58.7%)	
Employed	112	58 (51.8%)	54 (48.2%)	
**Radio ownership**				0.010
Yes	1,211	530 (43.8%)	681 (56.2%)	
No	357	129 (36.1%)	228 (63.9%)	

**Figure 2:**
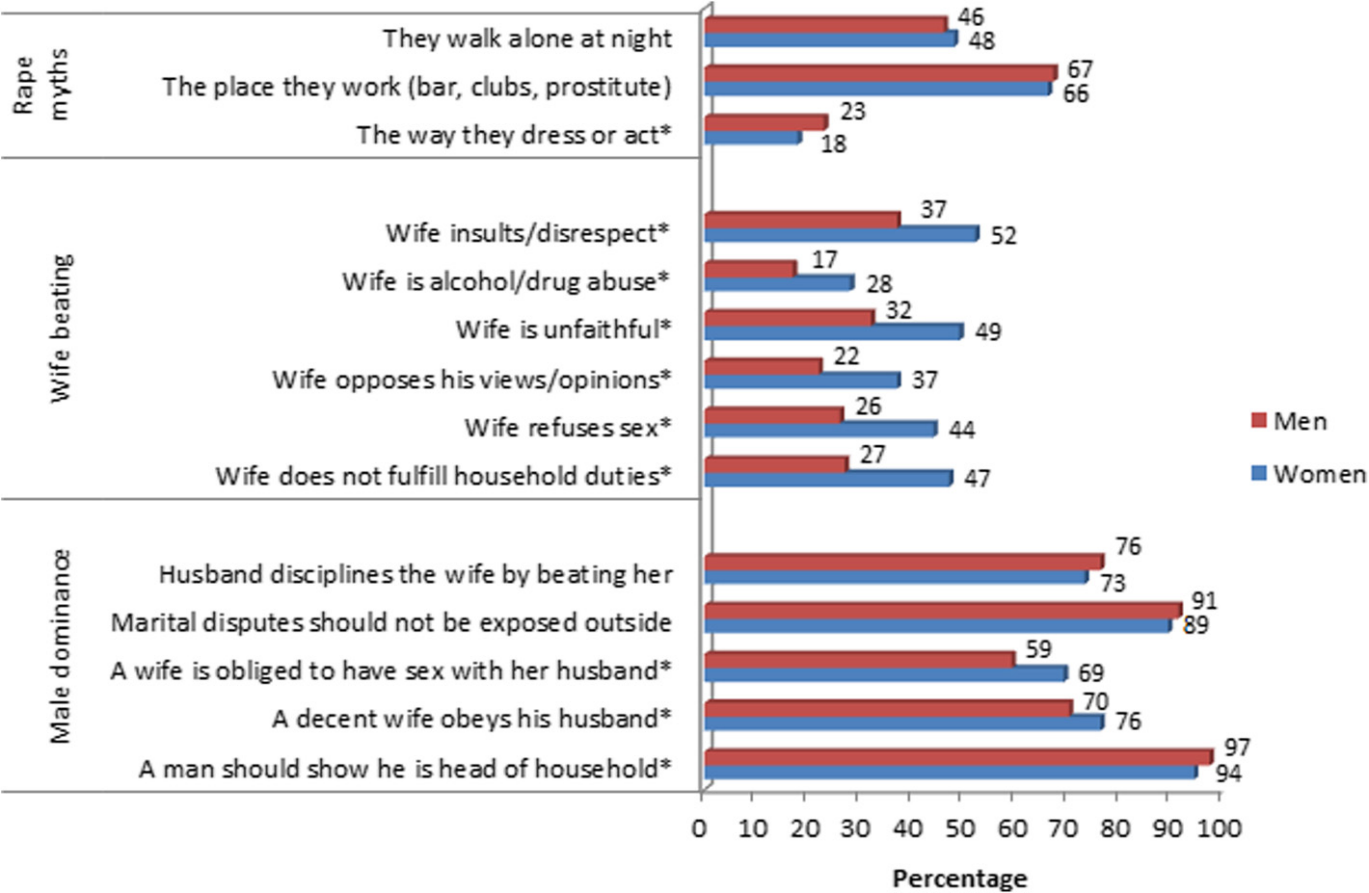
Gender analysis of attitude towards sexual violence. Percentage of all women and men aged 18–49 who believed that a husband is justified in beating his wife for the reasons given, who also endorsed the rape myths, and favored male dominance.

Figure 2 compares the norms and attitudes towards sexual violence by gender. Women were more likely to believe that men were justified in beating their wives for the majority of the reasons. Similarly, women were also likely to endorse the view that a woman or girl is raped because of the way they dress or act.

### Factors associated with attitudes to sexual violence

All the independent variables except for age showed a significant association with attitude towards sexual violence in the bivariate analysis. After adjusting for other variables, sex, age, education level and marital status persisted to show significant association. Men were more likely [AOR = 1.7 (95%CI: 1.4-2.1)] than women to express a non-accepting attitude towards sexual violence. Single and divorced groups were more likely to express a non-accepting attitude towards sexual violence [Single: AOR = 1.8 (95%CI: 1.4-2.3)]; [Divorced: AOR = 1.8 (95%CI: 1.3-2.6)] compared to the married group. The results for those with primary education were not significantly different from those who had never gone to school [AOR = 1.3 (95%CI: 0.9-2.0)]. The details are as shown in Table 6.

**Table 6:** **Bivariate and multivariate logistic regression analysis of attitude towards sexual violence among respondents of rural Morogoro**

**Predictors**	**Number (N = 1,568)**	**Non- accepting attitude n (%)**	Crude OR*	**95% confidence interval (CI)**	Adjusted OR**	**95% confidence interval (CI)**
**Sex**						
Female	915	331 (36.2%)	Ref		Ref	
Male	653	328 (50.2%)	1.8	(1.5-2.2)	1.7	(1.4-2.1)
**Age (in years)**						
15 – 24	410	162 (39.5%)	Ref		Ref	
25 – 34	595	243 (40.8%)	1.1	(0.8-1.4)	1.3	(1.0-1.7)
35 – 49	563	254 (45.1%)	1.3	(1.0-1.6)	1.5	(1.2-2.1)
**Educational level**						
No formal Education	142	46 (32.4%)	Ref		Ref	
Primary Education	1,308	542 (41.4%)	1.5	(1.0-2.1)	1.3	(0.9-2.0)
Secondary Education and above	118	71 (60.2%)	3.2	(1.9-5.2)	2.3	(1.3-4.0)
**Marital status**						
Married/cohabiting	969	358 (36.9%)	Ref		Ref	
Single	444	229 (51.6%)	1.8	(1.4-2.3)	1.8	(1.4-2.3)
Divorced/separated/widow	155	72 (46.5%)	1.5	(1.1-2.1)	1.8	(1.3-2.6)
**Occupation**						
Unemployed	1,456	601 (41.3%)	Ref		Ref	
Employed	112	58 (51.8%)	1.5	(1.0-2.2)	1.3	(0.9-1.9)
**Radio ownership**						
No	357	129 (36.1%)	Ref		Ref	
Yes	1,211	530 (43.8%)	1.4	(1.1-1.8)	1.3	(1.0-1.7)

OR - Odds Ratio.

*Bivariate logistic regression.

**Multiple logistic regression, age, sex, education, marital status, occupation and radio ownership were included.

## Discussion

Our study aimed at describing the rural communities’ knowledge and attitudes towards rape and child sexual abuse. The communities portrayed poor knowledge and significant accepting attitudes towards sexual violence. Gender of respondents, age, marital status and level of education were associated with knowledge and attitude towards sexual violence.

In Tanzania, women are disadvantaged compared to men in terms of education and earnings, factors that greatly influence the health of women and children. Overall, 19% of women aged 15–49 have received no formal education, almost twice the proportion of men (10%) [42]. In this study, the majority of participants had only completed primary education, and this factor could have contributed to their poor knowledge on the health consequences of sexual violence and its treatment, and the SOSPA law that convicts the perpetrators. This finding confirms what has been found previously in an urban setting in Tanzania [27-29]. The higher the level of education, the more likely the participants were to have good knowledge on sexual violence. This finding suggests that educational reform can prevent gender-based violence by empowering women through education, increasing school safety, and by promoting better attitudes and practices among students with regard to women’s human rights [49]. Improvement in the education program can be used as a primary strategy to stop, or at least decrease, the amount of sexual violence, which also has an impact on gender attitudes. It is likely to be successful if introduced at primary school level in order to make the school environment safer and to promote a better response to rape survivors from a large section of the population. In Tanzania, radio has substantial reach. Community level campaign efforts can be supported by using radio, as our findings have shown that those who had access to a radio had an increased likelihood of having higher knowledge on sexual violence.

In accordance with previous research, we found significant attitudinal differences by demographic factors: gender, age, education level and marital status. Non-accepting attitudes were prominent among participants with higher levels of education. Students’ values and attitudes are believed to become more liberal, egalitarian, and tolerant over the course of their college career [50]. In general, liberal and tolerant attitudes are highly correlated with rejection of rape myths and lower levels of victim blaming. In our sample, most men tended to be less supportive of gender stereotypes and rape myths than did women. Women who may be potential victims of violence justified and endorsed the rape myths. Our findings are in line with a study conducted in Uganda which showed that 70% of men and 90% of women viewed the beating of the wife or female partner as justifiable in some circumstances [26]. The results are also consistent with other studies that examined this association in Sub-Saharan Africa [37-39]. These observations pose a challenge in preventing sexual violence in such setting. The attitudes reported by men in this study may have been influenced by the current on-going project, “The Champion”, which focuses on involving ‘Men as Partners’ in addressing gender roles, reproductive health and in openly opposing intimate partner violence [51]. The project implemented a mass media and community-based communications campaign that aimed to reduce societal acceptance of GBV in Tanzania in 2011–2012. The campaign had a theme of *‘kuwa mfano wa* kuigwa’ (Be a Role Model). This significant difference between men and women’s acceptability of violence may be attributed to contextual factors such as women’s disempowerment, low educational and occupational status, poverty and rural residency [52-54]. Other research indicates that men are associated with greater likelihood of accepting rape myths and traditional gender stereotypes [33,55,56]. However, factors accounting for inequalities in men’s attitude towards sexual violence have not been adequately studied. Because societies may differ in terms of political, social, cultural and empowerment factors, a unique set of need-adapted interventions to suit the context of each society may be required.

We also found that as age increased, beliefs in rape myths and acceptance of sexual violence attitudes decreased. Older people of both genders were more likely to express non-accepting violence attitudes than did the younger age groups of below 25 years. Although age has been studied less than gender, race or education level, existing research shows that it is a salient demographic factor [57,58]. In this study, the younger participants were more likely to support rape myths or to blame the victim. This finding contradicts observations from other studies about attitudes towards sexual violence that suggest more accepting attitudes towards sexual violence are found in older people [50,57,58]. One plausible explanation for this difference could be that, in this community, sexual violence and other social disputes are still traditionally resolved under the authority of elders [28,59]. It is important therefore to recognize this attitude gap between these two generations and address interventions targeting different age groups in future studies.

The study design has several strengths and limitations worth discussing. We tried to minimize selection bias in a variety of ways. This was a large population- based study which adopted a random sampling method. Response rates were maximized in a number of ways: at least three repeat visits were made to households where respondents were not available at the time of the first visit. Although the nature of this topic was sensitive, several features are likely to have increased the validity of such reporting, including the training of interviewers on how to introduce the study and build rapport with respondents, the close interaction between interviewers and respondents, and efforts undertaken to ensure the privacy and confidentiality of responses. The cross-sectional design also suited our objective to measure the impact of community- rather than individual-level intervention. Most studies have measured attitude to rape by the acceptance of rape myths, societal gender stereotypes and interpersonal violence [55,56]. Our measure of attitudes towards rape and acceptability of violence using validated cross-cultural scales [5,43] ensured that findings are comparable with other studies because such interpretations of rape have been demonstrated to relate to acceptance of gender stereotypes and rape myths [32]. Despite these strengths, the study also has some potential limitations to our results. Our study relied on face-to-face interviews, in which, unlike in self-administered questionnaires, participants’ reported attitudes may not be only what they believe and know, but more what they have learned to respond, for example, what is socially acceptable, leading to response bias. This potential limitation could be overcome by including a measure of social desirability to account for such responses.

## Conclusions

In this rural community in Tanzania there is poor knowledge about sexual violence and, commonly, an accepting attitude towards sexual violence. Increasing age and higher education were associated with better knowledge and less accepting attitudes towards sexual violence. The study findings have potentially important implications for interventions aimed at preventing violence. The results highlight the challenges associated with changing attitudes towards sexual violence, considering that the highest levels of support for such violence were found among women. Incorporating education programs in schools and universities that focus on violence against women and children, and also using available mass media, might be an effective strategy for changing attitudes about rape and rape victims and promoting a better response to rape survivors. Moreover, it is important that these programs are tailored to suit this particular context by channeling them through the appropriate relationships, social institutions, gatekeepers, and community leaders.
